# TLR 9 (rs352140) gene polymorphism in *Helicobacter pylori* infection in children

**DOI:** 10.1038/s41598-026-58037-5

**Published:** 2026-06-26

**Authors:** Nashwa Farouk Mohamed, Ola Galal Ali Behairy, Hebatallah Emam Mohammed Ahmed, Fatma Abd ELHalim Mohamed Abd ELHalim

**Affiliations:** 1 Pediatrics, Faculty of Medicine, Benha University, Benha, 15131 Egypt; 2 Biochemistry, Faculty of Medicine, Benha University, Benha, Egypt

**Keywords:** TLR9, Gene polymorphism, *Helicobacter pylori*, Children, Diseases, Gastroenterology, Genetics, Medical research, Microbiology

## Abstract

Most of the global populace is susceptible to *Helicobacter pylori* (*H. pylori*) infection, which typically manifests in childhood. This study aimed to identify the role of TLR9 (rs352140) gene in suppressing or promoting the inflammation related to *H. pylori* infection in children. This cross-sectional study enrolled 100 children with dyspeptic symptoms undergoing upper endoscopy, including 50 with confirmed *H. pylori* infection and 50 age- and sex-matched H. pylori-negative controls. All children undertook full history, complete clinical investigation, laboratory testing, upper digestive endoscopies and genotyping of TLR9 (rs352140). A statistically significant difference presented among *H. pylori* positive and *H. pylori* negative children as regards TLR rs352140 gene polymorphism, as patients have statistically higher frequencies of homozygous TT genotype, CT genotype and of variant T allele in contrast to *H. pylori* negative group. Hazard of *H. pylori* is 7.9 times higher in children with TT genotype and 3.6 times higher in children with CT genotype in contrast to children carrying CC genotype. Also, children carrying the altered T allele had a statistically higher risk of *H. pylori*, 2.3 times in contrast to those carrying C allele. TLR9 rs352140 gene polymorphism was correlated with of *H. pylori* infection and incidence of gastritis in children.

## Introduction

Helicobacter pylori (H. pylori) infection is a major cause of chronic gastritis worldwide, with acquisition typically occurring during childhood and persisting for decades if untreated^1,2^. Beyond its immediate effects on gastric mucosa, chronic infection is associated with peptic ulcer disease and increased risk of gastric adenocarcinoma and mucosa-associated lymphoid tissue lymphoma later in life^3,4^. Current diagnostic gold standards include endoscopic biopsy with rapid urease testing and histopathology^5^.

Toll-like receptors (TLRs) are key mediators of innate immunity, recognizing pathogen-associated molecular patterns (PAMPs) and initiating inflammatory responses^6,7^. Among these, TLR9 is an endosomal transmembrane receptor that specifically recognizes unmethylated CpG dinucleotide motifs, which are abundant in bacterial DNA—including that of H. pylori^8^. Upon CpG DNA binding, TLR9 activates MyD88-dependent signaling pathways, leading to nuclear factor kappa B (NF-κB) activation and subsequent production of pro-inflammatory cytokines such as IL-8, IL-1β, and TNF-α^9,10^. In the human stomach, gastric epithelial cells express both TLR4 and TLR9, positioning TLR9 as a critical sentinel for bacterial DNA sensing^8,11^.

Genetic variation in TLR9 may therefore influence host susceptibility to H. pylori infection and the severity of resulting gastritis. The single nucleotide polymorphism (SNP) rs352140 (G2848A) is a synonymous variant located in exon 2 of the TLR9 gene. Although it does not alter the amino acid sequence, it may affect mRNA stability, splicing efficiency, or translational kinetics, potentially leading to dysregulated TLR9 expression or signaling^12,13^. Previous studies have reported conflicting associations between rs352140 and H. pylori-related outcomes across different populations, with some showing increased risk among variant carriers and others finding no significant association^14–16^.

Accordingly, this study aimed to investigate the association between the TLR9 rs352140 gene polymorphism and susceptibility to H. pylori infection in children, as well as its potential role in modulating the severity of gastritis in affected individuals.

## Patients and methods

This cross-sectional study was conducted on 100 children admitted at the Gastroenterology and Hepatology Unit in the Pediatric Department of Benha University Hospital, Al-Qalyubia, Egypt, for endoscopy (Approval No: MS-14-12-2022) during the period from January 2023 to September 2025).

Inclusion criteria of the case group were children with age between 1 and 18 years with gastrointestinal symptoms that necessitates upper digestive endoscopy like: persistent pain in the abdomen and/or the stomach, Heartburn, vomiting, and nausea.

Additionally, children who showed red flag signs like anemia, gastrointestinal hemorrhage, failure to thrive, or increased ESR were as well involved.

Exclusion criteria involved cases on proton pump inhibitors, those with substantial medical diseases, and those with gastrointestinal conditions that could explain their stomach discomfort (such as celiac disease, functional abdominal pain and inflammatory bowel disease).

Patients who had received proton pump inhibitors within 2 weeks, or antibiotics or bismuth compounds within 4 weeks prior to endoscopy were excluded to avoid false-negative results.

All children included in the study presented with dyspeptic symptoms (abdominal pain, heartburn, vomiting, nausea) and/or red flag signs (anemia, gastrointestinal hemorrhage, failure to thrive, elevated ESR) that warranted upper endoscopy. From a larger cohort of eligible patients undergoing endoscopy, 50 children with confirmed H. pylori infection (positive rapid urease test and confirmatory histopathology) were selected as the positive group. An additional 50 age- and sex-matched children with negative H. pylori status (negative by both rapid urease test and histopathology) were selected as the control group. This balanced 50:50 design was chosen to optimize statistical power for comparing genotype frequencies between groups.

All children underwent full history taking, laboratory investigations and complete clinical examination as following.

1. *Clinical evaluation*: Physical examination and full history were obtained. Body mass index (BMI), height, and weight, were all evaluated as portion of anthropometrics. Egyptian pediatric references were utilized to compute percentiles^17^.
2. *Radiological examination*: In an effort to identify structural and inflammatory conditions, abdominal ultrasound examinations were conducted with curved linear transducers that operated at frequencies between 2 and 5 MHz and linear transducers that operated at frequencies between 4 and 8 MHz (Xario XG; Toshiba, Tokyo, Japan).
3. *Laboratory analysis*: 3 ml sample was obtained in an ethylenediaminetetraacetic acid (EDTA) vial for a complete blood count (CBC). The analysis was conducted using a CELL-DYN Emerald automated hematology analyzer from Germany to measure hemoglobin (Hb), leukocytes, and platelets. ESR was measured by the Westergren method^18^, using 3 ml anti-coagulated blood allowed to separate under gravity for 1 h. Stool analysis examined color, consistency, and microscopy (1 gram sample). Stool culture to detect pathogenic bacteria. Urine analysis examined the chemical properties and sediments (5–10 ml midstream sample). Blood agar (1 ml urine) was employed to quantify colony forming units in urine culture. The qualitative immunochromatographic bioNexia^®^ reagent (0.15 g stool) was employed to conduct H. pylori stool antigen examination, which was predicated upon anti-H. pylori antibodies, following the manufacturer’s protocol^19^.
4. *Diagnostic upper gastrointestinal endoscopy*: Diagnostic upper gastrointestinal endoscopy was performed under general anesthesia with endotracheal intubation (for children < 10 kg or with risk factors for aspiration) or deep sedation with spontaneous ventilation. Intravenous access was secured, and standard monitoring (electrocardiography, non-invasive blood pressure, pulse oximetry) was applied. Anesthesia was induced with propofol (1–3 mg/kg) combined with fentanyl (1–2 mcg/kg) or remifentanil (0.3–0.5 mcg/kg). Midazolam (0.05–0.1 mg/kg) was used for anxiolysis. Anesthesia was maintained with sevoflurane (if intubated) or intermittent propofol boluses. Atropine (0.02 mg/kg) was administered as needed for secretions or bradycardia. It was performed using general anesthesia on a cohort of 100 children using an Olympus XQ20 gastroscope, following standard pediatric endoscopic guidelines^20^. At least six gastric biopsy samples were procured from the antrum, body, and duodenum during each procedure for histopathology H&E staining for detection of *H. pylori* and 1 biopsy for rapid urease and 1 biobsy for TLR9. These biopsies underwent formalin fixation, paraffin embedding, and subsequent examination through H&E and Giemsa staining to identify *H. pylori* presence. Additionally, the rapid urease test was administered on the biopsies using the commercially available Campylobacter-like Organism (CLO) kit (PYLO-PLUS, Gulf Coast Scientific, Oldsmar, FL, USA), with a change in color change from yellow to red within 60 min signifying a positive outcome, as previously described^21^. The nursing staff monitored the color transformation at predetermined intervals. Endoscopic assessments also encompassed the evaluation of bleeding and tissue damage. Histopathological analysis was performed to assess *H. pylori* density, chronic inflammation, atrophy, activity, and intestinal metaplasia according to the Updated Sydney System^22^. Parameters assessed included *H. pylori* positivity, histological grade of neutrophil infiltration (activity), mononuclear cell infiltration (chronic inflammation), glandular atrophy, and intestinal metaplasia. These parameters were scored; as null (0), mild^1^, moderate^2^, and severe^3^.

H. pylori positivity was defined by a positive rapid urease test (color change from yellow to red within 60 min) and confirmatory histopathology (Giemsa staining showing H. pylori organisms). H. pylori negativity required both tests to be negative.

5. *Genotyping of TLR9 (rs352140)*

A venous blood sample (2 mls) were withdrawn from each subject and were collected into sterile ethylene diaminetetra acetate “EDTA” (vacutainer) tube and were used for DNA extraction. DNA was extracted from fresh samples and stored at -20 °C till time of assay for determination of TLR9 (rs352140) polymorphism by using Real Time Polymerase chain reaction (RT-PCR) technique. The blood samples were used for DNA extraction using the Gene JET Whole Blood Genomic DNA purification Mini Kit (Thermo Fisher Scientific, Germany) according to the manufacturer’s protocol^23^. Determination of DNA concentration was done using Nanodrop One spectrophotometer (thermoscientific, USA). A concentration of (1 µg) from each sample was used for genotyping by rtPCR assay. Genotyping of the TLR9 (rs352140) polymorphism was performed using a TaqMan predesigned SNP genotyping assay (Applied Biosystems, USA) with TaqMan Universal PCR Master Mix (No AmpErase UNG) on an Applied Biosystems™ 7500Fast Dx Real-Time PCR System (Applied Biosystems, Waltham, MA, United States) following standard protocols^24^. The primer sequences used were forward primer [F5′-GCAGCACCCTCAACTTCACC-3′], reverse primer [R5′-GGCTGTGGATGTTGTTGTGG-3′]. The predesigned assay (C_2301954_20) was utilized for allelic discrimination.

### Sample size calculation

The sample size was estimated regarding this Eq. (25) also according to parallel researches^25^:

$${\mathbf{N}}{\text{ }} = {\text{ }}\left[ {{\mathbf{Z}}{\text{ }} \times {\text{ }}{\mathbf{Z}}{\text{ }} \times {\text{ }}{\mathbf{P}}{\text{ }} \times {\text{ }}{\mathbf{Q}}} \right]{\text{ }}/{\text{ }}\left[ {{\mathbf{E}}{\text{ }} \times {\text{ }}{\mathbf{e}}} \right]$$

Z = 1.96, P= Prevalence, Q = 1-P, E = 0.05.

The equations were used to determine the final sample size of 100 contributors, which consisted of 50 H. pylori-positive and 50 H. pylori-negative participants, with calculations for both precision and significance.

### Statistical analysis

The composed data were tabulated and analyzed by SPSS version 16 software (SpssInc, Chicago, ILL Company). Categorical data were expressed as number and percentages, Chi square (χ^2^) and Fisher’s exact tests were utilized to analyze them, odds ratio (OR) and the corresponding 95%CI were considered when appropriate. Quantitative data were tested for normality using Shapiro-Wilks test assuming normality at *P* > 0.05. Normally distributed variables were expressed as mean ±standard deviation and analyzed by Student “t"for 2 independent groups, Binary logistic regression analysis was used to identify the significant predictors of *H. pylori*. *P* ≤ 0.05 was regarded significant.

## Results

All 100 enrolled children presented with dyspeptic symptoms or red flag signs requiring endoscopy. Among these, 50 had confirmed *H. pylori* infection and 50 were H. pylori-negative, selected as described in methods.

The *H. pylori* positive group included 50 children (24 males and 26 females) and *H. pylori* negative group included 50 children (25 males and 25 females). There was no statistically significant difference among *H. pylori* positive patients and *H. pylori* negative cases as regards to their age, sex or main complain. However, *H. pylori* positive group had statistically higher frequency of rural area (68%) compared to H pylori negative group (48%), *p* = 0.004, Table 1.

**Table 1:** Sociodemographic data of the studied groups.

		*H. pylori* positive group		*H. pylori* negative group		Test	P value
		N = 50	%	N = 50	%		
Age (years)	Mean ± SD	10.4 ± 3.1		10.9 ± 3.5		t = 0.74	0.45
	Range	3–17		4–17			
Sex	Male	24	48.0%	25	50.0%	X^2^=0.43	0.84
	Female	26	52.0%	25	50.0%		
Residence	Rural	34	68.0%	24	48.0%	X^2^=3.1	0.004*
	Urban	16	32.0%	26	52.0%		
The main complain	Recurrent abdominal pain	47	94.0%	37	74.0%	X^2^=1.1	0.77
	Recurrent vomiting	20	40.0%	16	32.0%		
	Dysphagia	11	22.0%	14	28.0%		
	Hematemesis	3	6.0%	2	4.0%		

X^2^: Chi-square test, t: Student t-test.

*H. pylori* positive patients had statistically significant lower Hb (*p* = 0.004) and statistically higher NLR, ESR (*p* < 0.001 for both) and statistically higher frequency of positive *H. pylori* stool antigen (*p* < 0.001) in distinction with *H. pylori* negative group.

Children with *H. pylori* positive group had statistically increased frequencies of moderate, severe gastritis and duodenitis compared with children with negative *H. pylori* group (*p* < 0.001 and *p* = 0.032, respectively). While no statistical difference was observed among groups as regards esophagus examination. Regarding histopathological examination, A statistically significant difference was found among *H. pylori* positive and negative groups as regards to presence of active gastritis, polymorph nuclear cells and *H. pylori* activity (*p* < 0.001). None of patients in both groups had atrophy or metaplasia, Table 2.

**Table 2:** Upper endoscopic findings and Histopathological findings of studied patients.

		*H. pylori* positive group			*H. pylori* negative group		Test	P value
		N = 50	%		N = 50	%		
Endoscopic finding in esophagus	Normal	47	94.0%		48	48.0%	X^2^=0.65	0.76
	Esophagitis & esophageal erosion	3	6.0%		2	4.0%		
Endoscopic finding in stomach	Normal	0	0.0%		24	48.0%	X^2^=13.6	< 0.001*
	Diffuse mild gastritis	13	26.0%		14	28.0%		
	Moderate gastritis	23	46.0%		11	22.0%		
	Severe gastritis & nodularity	11	22.0%		1	2.0%		
	Gastric ulcers	3	6.0%		0	0.0%		
Endoscopic finding in duodenum	Normal	33	66.0%		45	90.0%	X^2^=4.9	0.032*
	Duodenitis	17	34.0%		5	10.0%		
Rapid urease test	Positive	50	100.0%		0	0.0%	–	–
	Negative	0	0.0%		50	100.0%		
Biopsy report of the stomach	Normal	0	0.0%		24	48.0%	X^2^=15.2	< 0.001*
	Mild chronic active gastritis	20	40.0%		14	28.0%		
	Moderate chronic active gastritis	17	34.0%		11	22.0%		
	Severe active gastritis	13	26.0%		1	2.0%		
Polymorph nuclear cells	Absent	10	20.0%		35	70.0%	X^2^=7.9	< 0.001*
	Present	40	80.0%		15	30.0%		
Atrophy	Absent	50	100.0%		50	100.0%	–	–
	Present	0	0.0%		0	0.0%		
Metaplasia	Absent	50	100.0%		50	100.0%	–	–
	Present	0	0.0%		0	0.0%		
*H. pylori* activity	No	0	0.0%		50	100.0%	X^2^=43.4	< 0.001*
	+	18	36.0%		0	0.0%		
	++	24	48.0%		0	0.0%		
	+++	8	16.0%		0	0.0%		

χ^2^: Chi-square test, *Statistically significant as p value < 0.05.

**Fig. 1:**
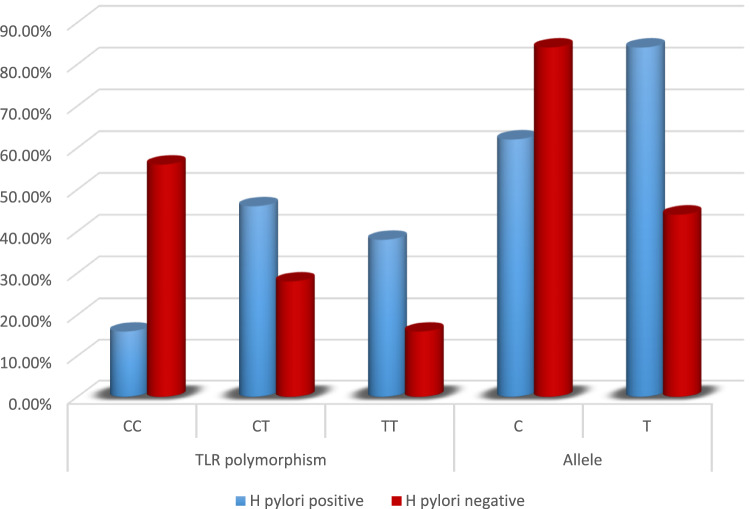
Comparison between the two studied groups regarding TLR-9 (rs352140) gene polymorphism.

There was statistically significant difference among *H. pylori* positive and *H. pylori* negative children as regards to TLR rs352140 gene polymorphism, as patients have statistically higher frequencies of homozygous TT genotype (38% vs. 16%, *p* < 0.001), CT genotype (46% vs. 28%, *P* = 0.031) and of variant T allele (84% vs. 44%, *p* = 0.027) in contrast to *H. pylori* negative group. The hazard of *H. pylori* is 7.9 times higher in children with TT genotype and 3.6 times higher in children with CT genotype compared to children carrying CC genotype. Also, children carrying the altered T allele had a statistically higher risk of *H. pylori*, 2.3 times in contrast to those carrying C allele, Fig. 1; Table 3.

**Table 3:** Comparison between the two studied groups according to TLR-9 (rs352140) gene polymorphism.

		*H.pylori* positive group		*H. pylori* negative group		OR 95% CI	P value
		N = 50	%	N = 50	%		
TLR genotypes	CC (Wild)	8	16.0%	28	56.0%	Reference	
	CT (Heterozygous)	23	46.0%	14	28.0%	3.6 (0.8–5.9)	0.031*
	TT (Homozygous)	19	38.0%	8	16.0%	7.9 (2.6–19.1)	< 0.001*
Allele	C	31	62.0%	42	84.0%	2.3 (1.1–5.9)	0.027*
	T	42	84.0%	22	44.0%		

R = reference genotype or allele, OR = odd ratio, CI = confidence interval, *: significant.

Children with TT genotype had statistically higher NLR (*p* = 0.019) and ESR (*p* = 0.025) compared to other genotypes, while there was no statistical difference between different genotypes as regarding to Hb, platelets or WBCs. Children with TT and CT genotypes had statistically higher frequencies of moderate severe active gastritis compared to CC genotypes (*p* = 0.041), while there was no statistical difference between different genotypes as regarding PMN, or H. pylori load, Table 4.

**Table 4:** Relation between TLR9 (rs352140) gene polymorphism and laboratory investigations, biopsy, histopathological examination of H pylori positive group.

				TLR9 polymorphism							Test		P value
				TT		CT			CC				
Hemoglobin (gm/dl)		Mean ± SD		10.5 ± 1.2		10.6 ± 1.6			10.7 ± 0.9		F = 0.22		0.91
		Range		8.4–11.9		9-12.3			9-11.8				
Platelets (10^3^/L)		Mean ± SD		295 ± 80		282 ± 82			309 ± 79		F = 0.57		0.56
		Range		193–441		214–400			193–420				
WBCs (10^3^/L)		Mean ± SD		7.7 ± 1.9		7.2 ± 2.3			6.9 ± 2.1		F = 0.32		0.73
		Range		5–11		4–11			4.6–11				
NLR		Mean ± SD		3.2 ± 1.2		2.6 ± 1.5			1.9 ± 1.2		F = 4.1		0.019*
		Range		0.8–5.2		0.9-5			0.8–5.1				
ESR		Mean ± SD		21.1 ± 5.2		13.6 ± 7.6			11.3 ± 5.5		F = 3.8		0.025*
		Range		4–45		5–51			3–47				
				N = 19	%	N = 23		%	N = 8	%			
Endoscopic finding in esophagus		Normal		18	94.7%	21		91.3%	8	100.0%	X^2^=1.6		0.80
		Esophagitis & esophageal erosions		2	10.6%	1		8.6%	0	0.0%			
Endoscopic finding in stomach		Diffuse mild gastritis		0	0.0%	5		21.7%	8	100.0%	X^2^=12.3		0.041*
		Moderate gastritis		11	57.9%	12		52.5%	0	0.0%			
		Severe gastritis & nodularity		6	31.6%	5		21.7%	0	0.0%			
		Severe gastritis & gastric ulcer		2	10.5%	1		4.3%	0	0.0%			
Endoscopic finding in duodenum		Normal		11	57.9%	15		65.2%	7	87.5%	X^2^=4.8		0.07
		Duodenitis		8	42.1%	8		34.8%	1	12.5%			
Biopsy report of the stomach		Mild chronic active gastritis		3	15.8%	11		47.8%	6	75.0%	X^2^=9.3		0.046*
		Moderate chronic active gastritis		9	47.4%	6		26.1%	2	25.0%			
		Severe active gastritis		7	36.8%	6		26.1%	0	0.0%			
Polymorph nuclear cells		Absent		2	10.5%	5		21.7%	3	37.5%	X^2^=2.6		0.19
		Present		17	89.5%	18		78.3%	5	62.5%			
Atrophy		Absent		19	100%	23		100.0%	8	100.0%	X^2^=0		–
		Present		0	0.0%	0		0.0%	0	0.0%			
Metaplasia		Absent		19	100%	23		100.0%	8	100.0%	X^2^=0		–
		Present		0	0.0%	0		0.0%	0	0.0%			
*H.pylori* activity		0		0	0.0%	0		0.0%	0	0.0%	X^2^=8.7		0.08
		+		5	26.3%	8		34.7%	5	62.5%			
		++		10	52.6%	12		52.2%	2	25.0%			
		+++		3	15.8%	4		17.4%	1	12.5%			

χ^2^: Chi-square test, *Statistically significant as p value < 0.05.

In univariate regression analysis, anemia, GI bleeding, failure to thrive, NLR, ESR, TLR-9 (rs352140) gene polymorphism TT and CT were significantly correlated with risk of *H.pylori* infection and incidence of gastritis. In multivariate regression model and only failure to thrive (OR: 2.9. *p* = 0.042), NLR (OR: 2.7, *P* = 0.036), and TLR-9 (rs352140) gene polymorphism TT (OR:6.7, *p* < 0.001) and CT (OR: 4.1, *p* = 0.039) were significantly associated to the risk of *H. pylori* infection and incidence of gastritis in the studied group, Table 5.

**Table 5:** Univariate and multivariate regression analysis of the factors correlated with the risk of H pylori infection and occurrence of gastritis.

	Univariate analysis		Multivariate analysis	
	OR (95% CI)	P value	OR (95% CI)	P value
Anemia	3.22 (1.11–10.73)	0.039*	1.87 (0.88–10.6)	0.09
GI bleeding	2.59 (1.15–24.8)	0.002*	1.96 (1.15–16.3)	0.12
Failure to thrive	4.36 (1.4–16.3)	0.015*	2.9 (0.52 − 7.89)	0.042*
NLR	2.3 (1.12–4.54)	0.012*	2.7 (1.1–5.34)	0.036*
ESR	1.9 (0.98–2.43)	0.041*	1.2 (0.87–4.23)	0.21
TLR-9 (rs352140) gene polymorphism TT	7.9 (2.6–19.1)	< 0.001*	6.7 (1–14.1)	< 0.001*
TLR-9 (rs352140) gene polymorphism CT	3.6 (0.8–5.9)	0.031*	4.1 (1.1–6.20	0.039*

*Statistically significant as p value < 0.05.

## Discussion

The saccharide backbone, a structural component, is utilized by TLR9, an endosome-transmembrane receptor, to recognize DNA in both damaged host cells and pathogens in an order-independent manner^26^. In a previous research^11^, In contrast to H. pylori-infected participants and healthy controls, the control group exhibited a preponderance of this TLR in the apical section of the gastric epithelium. In contrast, the TLR was primarily found in the basolateral section in H. pylori-positive individuals.

Based on our investigations a statistically significant difference was found among *H. pylori* positive and *H. pylori* negative children as regards to TLR rs352140 gene polymorphism, as patients have statistically higher frequencies of homozygous TT genotype (38% vs. 16%, *p* < 0.001), CT genotype (46% vs. 28%, *P* = 0.031) and of variant T allele (84% vs. 44%, *p* = 0.027) in contrast to *H. pylori* negative group. The hazard of *H. pylori* is 7.9 times higher in children with TT genotype and 3.6 times higher in children with CT genotype in contrast to children carrying CC genotype. Also, children carrying the altered T allele had a statistically elevated risk of *H. pylori*, 2.3 times unlike those carrying C allele.

Our results also agreed with *Loganathan et al.*^14^, , who studied TLR4 and TLR9 genetic variants as risk factors for chronic *H. pylori* infection in South Indian Tamils, on 77 patients, observed that TLR9 rs352140 heterozygous variant favored the perseverance of the H. pylori infection [*p* = 0.037, OR-1.87, 95% CI:1.07–3.29]. These results indicate that TLR9 rs352140 polymorphisms are possible genetic risk factors that affect the clinical manifestation and illness predisposition of chronic H. pylori infection in Indian Tamils.

While *Vaez et al.*^15^, , found that the genotype incidence of CC, CT, and TT for TLR9 rs352140 SNP was 43.4%, 42.6%, and 14% in H. pylori infection-negative participants, and 32.5%, 59.7%, and 7.8% in H. pylori infection-positive participants. Furthermore, the frequencies of C and T alleles were 62.3% and 37.7% in participants with H. pylori infection, and 64.8% and 35.2% in participants without H. pylori infection. The incidence of the variant allele (T) was 35.2% in H. pylori infection-negative participants and 37.7% in H. pylori infection-positive participants, while the combined frequency of variant genotypes (CT and TT) was 56.6% in negative participants and 67.5% in positive participants. Their findings indicated a substantial correlation between exposure to H. pylori infection and the TLR9 rs352140 CT variant genotype.

In contrast to *Meli et al.*^13^, , who observed that the control group had 58.88% CT, 18.69% TT, and 22.43% CC, with no substantial variances (*P* = 0.1075).

Conversely, heterozygotes CT comprised 43.75% of the participants, homozygotes TT comprised 33.33%, and homozygotes CC comprised 21.92%.

The disparities among the studies can be accredited to the genetic diversity among various ethnic groups. This variation may be linked to an elevated presentence of TLR9 and an elevated incidence of B cells + IgM. The release of pro-inflammatory cytokines, involving IL8, IL1β, TNF-α, and Type I interferons, is induced by the engagement of TLR9 ligand. This, in turn, reduces the secretion of gastric acid, thereby promoting the perseverance of H. pylori and providing a stimulus for gastric metaplasia^27^.

The observed association between the TLR9 rs352140 variant T allele and more severe gastritis raises the question of the underlying biological mechanism. Although rs352140 is a synonymous polymorphism (G2848A) located in exon 2 that does not alter the TLR9 amino acid sequence, several non-coding mechanisms could explain its functional impact. One possibility is that the variant affects TLR9 expression levels. Synonymous single nucleotide polymorphisms (sSNPs) can influence mRNA stability, secondary structure, or translation efficiency by altering codon usage bias or disrupting exonic splicing enhancers (ESEs)^28,29^. Increased TLR9 expression in gastric epithelial cells of T allele carriers could lead to heightened sensitivity to H. pylori CpG DNA, resulting in exaggerated downstream signaling. Alternatively, the variant might alter the conformation or dynamics of the nascent TLR9 polypeptide during translation (a phenomenon known as “cotranslational folding”), potentially affecting protein trafficking, dimerization, or ligand-binding affinity^30^. A more direct mechanism involves the TLR9 signaling cascade itself. Upon CpG DNA binding, TLR9 recruits the adaptor protein MyD88, leading to activation of NF-κB and mitogen-activated protein kinases (MAPKs), which drive transcription of pro-inflammatory cytokines including interleukin-8 (IL-8), tumor necrosis factor-alpha (TNF-α), and interleukin-1 beta (IL-1β)^9,10^. Variant-associated dysregulation—whether through increased TLR9 expression, enhanced receptor signaling, or impaired negative feedback loops could result in excessive or prolonged production of these cytokines. Elevated IL-8 is a potent neutrophil chemoattractant and has been directly implicated in H. pylori-associated gastric mucosal injury^31^. Our finding that TT genotype carriers had significantly higher NLR and ESR is consistent with a pro-inflammatory phenotype characterized by enhanced neutrophil recruitment and systemic inflammation. Future functional studies measuring TLR9 mRNA and protein expression, as well as cytokine profiles (IL-8, TNF-α, IL-1β) in gastric biopsies stratified by rs352140 genotype, are needed to distinguish between these mechanisms.

The issue of controversial has been the development of H. pylori infection findings regarding the function of TLR9. Consequently, a research conducted in murine models demonstrated the use of TLR9 in identification of DNA of H. pylori induces proinflammatory actions^32^, while the investigation conducted by *Otani et al.*^33^. conducted on mice suggested the receptor may serve as an inhibitor for H. pylori infection throughout the acute stage. According to, *Varga and Peek*^34^ It was suggested that TLR9 may have a controversial function, and its ability to suppress or promote may be predisposed by the gastric microenvironment. Consequently, the microenvironment, which is characterized by inflammatory cells lacking polarity, serves as a catalyst for the activation of proinflammatory forces through TLR9, which ultimately leads to the growth of gastric cancer. Additionally, TLR9 expression is upregulated in cancerous tissue^35^.

In the current work, Children with TT genotype had statistically higher NLR and ESR compared to other genotypes, while there was no statistical difference between different genotypes as regarding to Hb, platelets or WBCs. Children with TT and CT genotypes had statistically higher frequencies of moderate severe active gastritis compared to CC genotypes, while there was no statistical difference between different genotypes as regarding PMN, or *H. pylori* load.

Similarly, *Zhang et al.*^12^, identified a significant correlation among the TT genotype of TLR9 rs352140 SNP and the total effect of neoplasia, indicating that the polymorphism could potentially affect innate immune responses, thereby endorsing chronic inflammation and consequent carcinogenesis. Another study by *Eed et al.*^16^, evaluated persistent gastritis, peptic ulcer illness, and gastrointestinal cancer cases, which indicated that the TT genotype was predominantly present in the chronic gastritis group.

This finding was also shown in the study by *Meli et al.*^13^, , who evaluated the purpose of this SNP chronic gastritis children caused by H. pylori and demonstrated that the TT genotype of TLR9 rs352140 polymorphism was substantially related to increased levels of leukocytes and neutrophils.

The discrepancy among the absence of a variance in the distribution of TLR9 rs352140 genotype among both groups and the abovementioned significant positive correlation among the TT genotype of this polymorphism and elevated leukocytes and neutrophils may become attributed to the possibility that people carrying this genotype may prompt elevated values of the parameters because of specific trigger. An additional potential clarification could be associated with the comparatively small sample size of the work. In contrast, the low number of subjects may have influenced a substantial correlation among the TT genotype and the growth in leukocytes and neutrophils together. However, this poses a serious issue in this zone that necessitates additional research on bigger samples.

However, *Loganathan et al.*^14^, , conducted a study in grown-ups from India and found that the heterozygous CT genotype of this polymorphism is substantially related to perseverance of H. pylori infection.

In the current study, in univariate regression analysis, anemia, GI bleeding, failure to thrive, NLR, ESR, TLR-9 (rs352140) gene polymorphism TT and CT were significant predictors of risk of *H. pylori* infection and incidence of gastritis. In the studied cases. In multivariate regression model and only failure to thrive, NLR, and TLR-9 (rs352140) gene polymorphism TT and CT were significant predictors of risk of *H. pylori* infection and occurrence of gastritis in the studied group.

In systemic lupus erythematous, bacterial meningitis, renal transplantation, main immune thrombocytopenia, cervicitis, placental inflammation, or cervical cancer cases, the function of TLR9 rs352140 polymorphism was evaluated^36^. Nevertheless, there is a scarcity of data regarding gastropathy and the TLR9 rs352140 SNP.

TLR9 is an intracellular receptor that contributes to immune identification and signalling after *H. pylori* infection by identifying unmethylated CpG oligonucleotides in DNA. TLR9 signalling pathway has anti-inflammatory effects as a mean of maintaining homeostasis and is crucial for protecting the gut and healing from injuries. Therefore, the changes in the TLR9 may affect the course of the *H. pylori* associated gastric diseases. TLR9 gene polymorphism may cause altered expression and dysregulation of the TLR9 signalling that lead to disparity between pro- and antiinflammatory cytokine responses with unbalanced formation of inflammatory cytokines that can causes chronic inflammation^37^.

Our study is limited by the relatively small sample size (100 children), which is reflected in the wide confidence intervals for some odds ratios (e.g., TT genotype OR: 7.9, 95% CI: 2.6–19.1). Additionally, findings from a single Egyptian center may not be generalizable to other ethnic populations.

## Conclusion

Our results identified a stronger correlation between the TLR9 rs352140 gene polymorphism and the hazard of H. pylori infection and the incidence of gastritis in children. Additionally, they underscored that TT genotype carriers of the TLR9 rs352140 gene polymorphism may exhibit an additional serious grade of inflammation. However, additional investigation is necessary to determine the accurate function of innate immunity and its TLR9 polymorphisms in the progress of H. pylori gastritis in larger samples of pediatric cases.

## Data Availability

Deidentified individual participant data (including data dictionaries) will be made available, in addition to study protocols, the statistical analysis plan, and the informed consent form. The data will be made available upon publication to researchers who provide a methodologically sound proposal for use in achieving the goals of the approved proposal. Proposals should be submitted to [nashwafarouk16@gmail.com](mailto: nashwafarouk16@gmail.com). The data is available on reasonable request from the authors.

## Abbreviations

*H. pylori*: *Helicobacter pylori*
NK cells: Natural killer cells
TLRs: Toll-like receptors
PAMPs: Pathogen associated molecular patterns
CBC: Complete blood count
EDTA: Ethylenediaminetetraacetic acid
Hb: Hemoglobin
ESR: Erythrocyte sedimentation rate
CLO: Campylobacter-like organism
RT-PCR: Real time polymerase chain reaction
SNPs: Single nucleotide polymorphisms
NLR: Neutrophil/Lymphocyte ratio
